# Pravastatin and placental insufficiency associated disorders: A systematic review and meta-analysis

**DOI:** 10.3389/fphar.2022.1021548

**Published:** 2022-11-09

**Authors:** Ayala Hirsch, Reut Rotem, Natali Ternovsky, Bruria Hirsh Raccah

**Affiliations:** ^1^ Department of Obstetrics & Gynecology, Shaare Zedek Medical Center, Affiliated with the Hebrew University School of Medicine, Jerusalem, Israel; ^2^ Division of Clinical Pharmacy, Institute for Drug Research, School of Pharmacy, Faculty of Medicine, Hebrew University of Jerusalem, Jerusalem, Israel; ^3^ Department of Cardiology, Hadassah University Hospital, Jerusalem, Israel

**Keywords:** statins, pravasatin, preeclampsia, IUGR, uteroplacental, fetal growth restriction

## Abstract

**Background:** Uteroplacental insufficiency associated disorders, such as preeclampsia, fetal growth restriction and obstetrical antiphospholipid syndrome, share pathophysiology and risk factors with cardiovascular diseases treated with statins.

**Objective:** To evaluate pregnancy outcomes among women with uteroplacental insufficiency disorders who were treated with statins.

**Search Strategy:** Electronic databases were searched from inception to January 2022

**Selection Criteria:** Cohort studies and randomized controlled trials.

**Data collection and analysis:** Pooled odds ratios were calculated using a random-effects model; meta-regression was utilized when applicable.

**Main Results:** The analysis included ten studies describing 1,391 women with uteroplacental insufficiency disorders: 703 treated with pravastatin and 688 not treated with statins. Women treated with pravastatin demonstrated significant prolongation of pregnancy (mean difference 0.44 weeks, 95%CI:0.01–0.87, *p* = 0.04, I^2^ = 96%) and less neonatal intensive care unit admissions (OR = 0.42, 95%CI: 0.23–0.75, *p* = 0.004, I^2^ = 25%). In subgroup analysis, prolongation of pregnancy from study entry to delivery was statistically significant in cohort studies (mean difference 8.93 weeks, 95%CI:4.22–13.95, *p* = 0.00) but not in randomized control studies. Trends were observed toward a decrease in preeclampsia diagnoses (OR = 0.54, 95%CI:0.27–1.09, *p* = 0.09, I = 44%), perinatal death (OR = 0.32, 95%CI:0.09–1.13, *p* = 0.08, I^2^ = 54%) and an increase in birth weight (mean difference = 102 g, 95%CI: -14–212, *p* = 0.08, I^2^ = 96%). A meta-regression analysis demonstrated an association between earlier gestational age at initiation of treatment and a lower risk of preeclampsia development (R^2^ = 1).

**Conclusion:** Pravastatin treatment prolonged pregnancy duration and improved associated obstetrical outcomes in pregnancies complicated with uteroplacental insufficiency disorders in cohort studies.

**Systematic Review Registration:** https://www.crd.york.ac.uk/prospero/ identifier CRD42020165804 17/2/2020.

## Introduction

Preeclampsia (PET) and fetal growth restriction (FGR) are serious complications of pregnancy that increase the morbidity and mortality of both foetuses and parturients. Both, PET and FGR, may be a result of placental insufficiency (Chaddha et al., 2004). additionally, abnormal placentation and its associated disorders may be encountered among women with obstetric antiphospholipid syndrome (APLAS); 20%–30% of pregnancies with APLAS are complicated with PET, FGR or foetal losses (Abou-Nassar et al., 2011). The spectrum of uteroplacental insufficiency disorders results from various factors, including poor trophoblast uterine invasion early in pregnancy, impaired transformation of the uterine spiral arteries to high capacity-low impedance vessels, abnormalities in the development of chorionic villi, endothelial dysfunction and pathologic changes in the antiangiogenic environment. (Gaiser, 2005; Ilekis et al., 2016) PET or FGR occurring early in the course of pregnancy might prompts early delivery, which can lead to neonatal death and disability arising from prematurity, in addition to maternal morbidity which may be associated with PET.

The role of statins in treating and preventing cardiovascular diseases is well established (Brugts et al., 2009); nonetheless, their potential benefit in treating multiple non-cardiovascular conditions is currently under investigation (He et al., 2018). Specifically, endothelial dysfunction in PET, FGR and APLAS is associated with abnormalities in lipid profile, high levels of triglycerides and oxidative stress (Roberts and Cooper, 2001). Statins inhibit HMG-CoA reductase, leading to reduced plasma cholesterol levels (Tsujita, 1990). Statins also have antioxidant, anti-inflammatory, anti-thrombogenic and vasodilating effects (Laufs et al., 1997; Wolfrum et al., 2003; Jain and Ridker, 2005; Bu et al., 2011; Violi et al., 2013). Statins regulate sFlt-1 which is a key antiangiogenic factor associated with the development of preeclampsia through its affect on Hmox-1 (Cudmore et al., 2007), the preclinical evidence supporting the use of statins in the treatment of PET was demonstrated in several animal models (Saad et al., 2014).

Current clinical knowledge regarding the efficacy of statins in the treatment of PET is inadequate. Individual studies presented contradicting results (Gajzlerska-Majewska et al., 2018). Accordingly, current guidelines do not recommend the usage of statins in the prevention or treatment of uteroplacental insufficiency disorders (ACOG Practice Bulletins, 2020).

In this systematic review and meta-analysis, we aimed to assess pregnancy outcomes associated with pravastatin treatment, in cases of primary prevention (pregnancies at high risk of development of uteroplacental insufficiency disorders) and secondary prevention (pregnancies complicated with PET/FGR/APLAS).

## Materials and methods

### Search strategy

This systematic review followed the Preferred Reporting Items for Systematic Reviews and Meta-Analyses (2020) framework guidelines (PRISMA) (Page et al., 2021) and the Meta-analysis Of Observational Studies in Epidemiology (MOOSE) guidelines. (Supplementary Table S1, S2) (Stroup et al., 2021).

We conducted three systematic database searches: the first included articles from 1953 to February 2020, the second included articles published from the last search to April 2021 and the third included articles published from the last search to January 2022. The search included PubMed/Medline, Embase, Clinical Trials Registry Clinicaltrials.gov and The Cochrane Library. Language restrictions were not set.

The search strategies incorporated index terms (Mesh) and free text words for the search concepts: pravastatin, atorvastatin, rosuvastatin, pregnancy combined by “AND”; and in each domain, the terms were combined by “OR” (Supplementary Table S3) The first domain contained terms on statins (including synonyms and abbreviations such as HMG-CoA reductase inhibitors), the second domain related to pregnancy.

The detailed protocol is documented online in the International Prospective Register of Systematic Reviews Registry (CRD42020165804). Because this study was a review and meta-analysis, Helsinki board approval was waived.

### Data sources and searches

In the search strategy, we included randomized controlled trials (RCTs), non-randomized controlled clinical trials, prospective and retrospective comparative cohort studies, and case-control studies. Every study that included women treated with statins during pregnancy was analysed. Duplicated reports, case reports, case series, cross-sectional studies, pharmacokinetic studies in healthy adults, animal studies, reviews, expert opinion, editorials, letters to the editor, comments, and studies with a high risk of bias were excluded.

### Study selection and data extraction

Two investigators (AH and NT) independently identified and extracted articles for potential inclusion, using the Rayyan QCRI web application for systematic reviews (Ouzzani et al., 2016). Disagreements were resolved by referral to a third reviewer (B.H.R.). The full texts of the resulting references were retrieved and analysed. If more than one study published data from the same cohort, we included the report with the higher quality according to the Risk of bias In Non-randomized Studies of Interventions (ROBINS-I) tool (Newcastle-Ottawa Quality Assessment Scale- NOS) to avoid overlap (GA Wells et al., 1932).

Exposure to statins during pregnancy was defined as exposure to any dose and in any trimester of pregnancy. Pregnancies that were defined as high risk for future development of uteroplacental insufficiency (Primary prevention) disorders and those that had developed uteroplacental insufficiency disorders (Secondary prevention) were included. The spectrum of uteroplacental insufficiency disorders included: PET, early-onset FGR (<28w), and women with APLAS who developed PET or FGR. Multiple pregnancies were excluded in patient-level data to avoid bias. The primary outcome included prolongation of pregnancy from study entry to delivery. The secondary outcomes included neonatal outcomes such as admission to the neonatal intensive care unit (NICU), birth weight and perinatal death, and the maternal occurrence of a new diagnosis of PET. Perinatal death was defined as stillbirth or death in the first month of life. Data were extracted from the included studies by a single reviewer and subsequently evaluated by the second reviewer. For studies that did not report the outcomes, we contacted the authors and requested the missing data.

### Quality assessment and the risk of bias

The risk of bias and the quality of the observational studies were assessed using the Newcastle-Ottawa Quality Assessment Scale (NOS) (GA Wells et al., 1932). The scale is based on eight criteria and provides a score ranging from 0 (high risk of bias) to 9 stars (low risk of bias). A 5-star rating and below was designated high risk of bias, six to seven stars intermediate risk of bias, and eight to nine stars low risk of bias. Randomized controlled studies were evaluated by the Cochrane Collaboration’s Risk of Bias Tool (Higgins et al., 2011). Summary assessments of the risk of bias were derived for each study. Assessments were carried out independently by two investigators (AH and NT).

### Data synthesis and analysis

Meta-analysis and meta-regression were performed using Comprehensive Meta-Analysis software. Random-effect pooled odds ratios (ORs) were calculated with the corresponding 95% confidence intervals (CIs) to summarize the results overall and within subgroups. Heterogeneity was assessed by using the I^2^ statistic. We performed subgroup analyses by study type (RCT, cohort). Random-effects meta-analysis was used to pool analyses. Meta-regression analyses were performed to evaluate whether differences in pravastatin dosage and timing of treatment initiation modified the association between exposure to pravastatin and outcomes, and to explain the heterogeneity in the estimated effect size. The data were analyzed using the Comprehensive Meta-Analysis software.

### Publication bias

Assessment of publication bias by visual inspection of the funnel plot was planned, provided the analysis would include at least ten studies.

## Results

### Study selection

**FIGURE 1:**
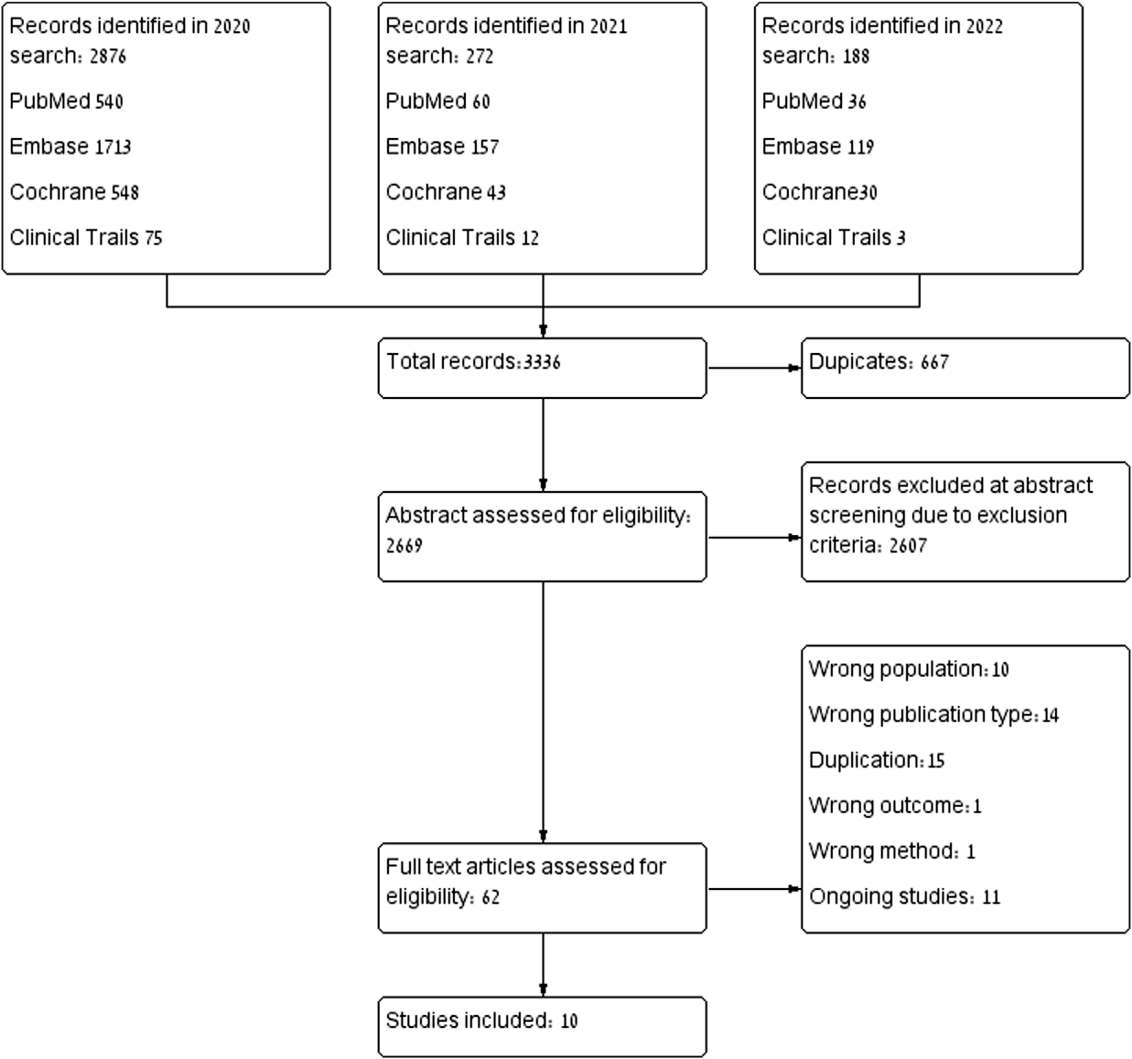
Publication selection process.

The database search yielded 3,336 citations: PubMed (n = 636), Embase (n = 1989), Cochrane Library (n = 621), clinicaltrials.gov (n = 90). In total, 667 were identical duplicates, and excluded. After abstract assessment, 62 articles were extracted for full-text review. Ten articles dropped out because they included normal pregnancies without any risk factors, nor signs or symptoms that indicated placental insufficiency. (Ofori et al., 2007; Taguchi et al., 2008; Colvin et al., 2010; Winterfeld et al., 2013; Bateman et al., 2015; McGrogan et al., 2017; Botha et al., 2018; Lee et al., 2018). One study that had examined only long-term outcomes was excluded because there were no other studies that examined the same outcome (Costantine, 2022) and another was excluded due to a lack of an appropriate control group (Kupferminc et al., 2021). Fourteen articles were excluded due to publication type or study design (Caroli-Bosc et al., 2001; Pollack et al., 2005; Toleikyte et al., 2011; RuysTitia et al., 2014; Daud et al., 2017; Desai et al., 2017; Kadioglu et al., 2020; Akbar et al., 2021). Studies involving animals or human placenta were excluded, due to a lack of standardization in studies investigating biomolecular markers, those studies or results were excluded as well, finally, only clinical studies were included. The selection process is illustrated in Figure 1. Ultimately, ten studies were included in the analysis, with a total of 1,391 pregnant women. Of these, 703 (50.5%) were treated with pravastatin and 688 (49.5%) were not treated with statins. All ten studies described high treatment adherence.

### Characteristics of the studies

The studies were published between 2016 and 2021 and originated from the United States (n = 2), Indonesia (n = 2), Serbia (n = 2), United Kingdom (n = 1), Spain (n = 1), Greece (n = 1), and multicentre over Europe (n = 1). Four (Lefkou et al., 2016; Lefkou et al., 2020; Mendoza et al., 2020; Jurisic et al., 2021) were cohort studies and six were RCTs (Costantine et al., 2016; Ahmed et al., 2020; Deviana et al., 2020; Dobert et al., 2021; Akbar et al., 2021; Costantine et al., 2021). Studies included women with uteroplacental insufficiency defined as early onset of PET between gestational age 24 and 31 weeks (Lefkou et al., 2016) (Ahmed et al., 2020) (Lefkou et al., 2020), early onset of FGR (Mendoza et al., 2020) (secondary prevention, n = 4) or high risk for developing PET (primary prevention, n = 6). In the primary prevention group, high risk was defined differently; in two studies as a history of severe PET in a prior pregnancy that required delivery before 34 weeks gestation (Costantine et al., 2016), (Costantine et al., 2021), in another study, as a history of previous PET and birth before 37 weeks of gestation or at least two main risk factors for PET (Deviana et al., 2020). An additional study defined high risk pregnancy as having a past poor obstetric history and abnormal placental Dopplers (Jurisic et al., 2021). Abnormal placental Dopplers were defined as uterine artery pulsatility index above the 95th percentile. One study selected women with at least 20% risk for developing PET based on the presence of minimally 2 independent clinical risk factors or abnormal doppler velocimetry index (Akbar et al., 2021) and another (Dobert et al., 2021) selected women with high risk for PET based on a survival-time model of Bayes theorem (Wright et al., 2020). In the secondary prevention group, two studies defined uteroplacental insufficiency as a pregnancy with an APLAS that developed PET or FGR associated with abnormal placental Dopplers (Lefkou et al., 2016; Lefkou et al., 2020) and one study included early onset of PET without proven APLAS (Ahmed et al., 2020). One study also included pregnant women with an early onset of FGR, diagnosed earlier than 28 weeks of pregnancy (Mendoza et al., 2020).

Although the search algorithm included all types of statins, only pravastatin was used in the studies included. Dosing: The dosage of pravastatin differed between studies: 40 mg in five studies, 20 mg in four studies and 10 mg in one study. The comparator treatment included aspirin together with low molecular weight heparin in two studies, aspirin alone in one study, placebo in four studies and no treatment in two studies. Timing of pravastatin initiation: Most studies initiated treatment with pravastatin in the second trimester, but in two studies a minority of the patients were diagnosed and started treatment in their third trimester (Lefkou et al., 2016; Ahmed et al., 2020) and one large RCT included only women in their late third trimester (Dobert et al., 2021). Table 1 displays a summary of the key characteristics of the included RCTs and cohort studies.

**TABLE 1:** Methodologic characteristics of the included studies.

Study, year, country, type	Study period	Intervention vs. control group	Indication for treatment	Initiation of treatment, weeks (median/mean, IQR)	Number of women pravastatin/control	Primary and secondary outcome measures
Ahmed, 2019, United Kingdom, RCT	2011–2014	Pravastatin 40 mg/Placebo	Early onset of PET	28w (mean) 24–30w	30/32	*Length of pregnancy
						*Perinatal death
						*Birth weight
						*NICU
						*Congenital malformation
						*PET features
						*sFlt-1 level
Lefkou, 2016, Greece, Cohort	2013–2015	Pravastatin 20 mg + Aspirin 80 mg + LMWH 40 mg/Aspirin 80 mg + LMWH 40 mg	APLS with FGR/PET	22w (median), w21-30	11/10	* Length of pregnancy
						*Perinatal death
						*Birth weight
						*PET features
Costantine, 2016, US, RCT	2012–2014	Pravastatin 10 mg/Placebo	High risk for PET	14w (median), 12–16w	10/10	*Drug side effects
						*Congenital anomalies
						*Preterm delivery
						*Perinatal death
						*Birth weight
						*PET features
						* sFlt-1 levels
Mendoza, 2020, Spain, Cohort	2016–2017	Pravastatin 40 mg/none	Early onset of FGR	24.4w (mean), 20–28w	19/19	* Length of pregnancy
						*Birth weight
						*PET features
						* sFlt-1/PlGF ratio
Jurisic, 2020, Serbia, Cohort	2015–2018	Parvastatin40 mg + arginine 1.5 g L/no treatment	abnormal doppler	w (mean),20.5 17–22w	10/5	* Foetal growth
						*PE features
						*Preterm delivery
						*Perinatal death
						*Birth weight
						NICU*
Lefkou, 2020, Serbia, Cohort	2016–2018	Pravastatin 20 mg + LMWH 40 mg + Aspirin 80 mg/LMWH 40 mg + Aspirin 80 mg	APLS with abnormal uterine Doppler	w (median), 24 w26-22	7/4	* Foetal growth
						*PET features
						*Length of pregnancy
						*Preterm delivery
						*Perinatal death
						*Birth weight
						NICU*
Soraya Riu, 2019, Indonesia, RCT	2018	Pravastatin 20*2 + Aspirin 80 mg/Aspirin 80 mg	High risk for PET	w 12–20 (no mean/median available)	18/15	* Foetal growth
						*PET features
						*Preterm delivery
Costantine, 2021, US, RCT		Pravastatin 20*1/Placebo	High risk for PET	14w 12–16+6w	10/10	Congenital malformations*
						*Medication side effect
						*Pharmacokinetic
						* Foetal growth
						*PET features
						*Preterm deliveries
						*Birthweight
						*NICU
Döbert, 2021, England, Spain, and Belgium, RCT	2018–2019	Pravastatin 20*1/Placebo	High risk for PET	35.9 (median) (35.4–36.1)	548/543	*Pet occurrence
						*Gestational hypertension
						*Perinatal death
						*Birth weight
						*Abruptio
						Neonatal morbidity. *
Akbar, 2021, Indonesia, RCT	2017–2020	Pravastatin 20 mg*2 + calcium 1 g + aspirin 80 mg/calcium 1 g + aspirin 80 mg	High risk for PET	15 (mean), 14–20	40/40	*Pet occurrence
						*PET features
						*Preterm deliveries
						*Birthweight
						*NICU
						* sFlt-1 levels, PlGF ratio

RCT-randomized controlled trial, LMWH-low, molecular weight heparin, PET-preeclampsia, FGR-intrauterine growth restriction, APLS-antiphospholipid syndrome, NICU-neonatal intensive care unit.

### Quality assessment

The overall risk of bias among the four nonrandomized studies according to the Newcastle-Ottawa Quality Assessment Scale (NOS) was 7.5. The overall risk of bias of the six randomized controlled studies evaluated by the Cochrane Collaboration’s Risk of Bias Tool was low. The risk of bias assessment is summarized in Supplementary Figures S1, S2.

As ten studies were included in the meta-analysis, we were able to test for funnel plot asymmetry to assess possible publication bias (Higgins et al., 2019). Visual inspection of the funnel plots revealed no indication of publication bias, Egger test also indicates no statistical significance in asymmetry (*p* = 0.5). (Supplementary Figures S3).

### Synthesis of results

#### Pregnancy prolongation

**FIGURE 2:**
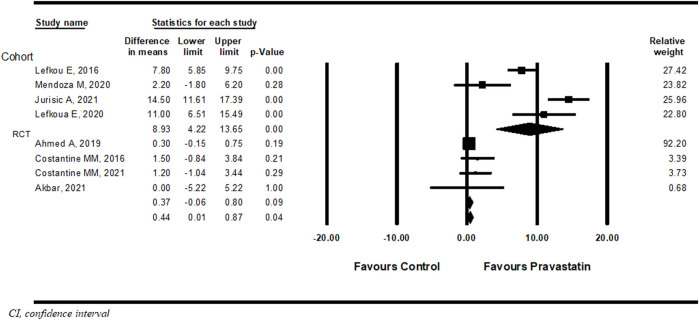
Meta-analysis results of the association of pravastatin treatment with a mean difference (weeks) in prolongation of pregnancy from study entry to delivery.

Eight studies (Costantine et al., 2016; Lefkou et al., 2016; Ahmed et al., 2020; Lefkou et al., 2020; Mendoza et al., 2020; Akbar et al., 2021; Costantine et al., 2021; Jurisic et al., 2021) compared the difference in prolongation of pregnancy from study entry to delivery between women treated with pravastatin and women not treated. Pravastatin treatment was associated with a significant prolongation of pregnancy from study entry to delivery of 0.44 weeks (mean difference 0.44 weeks, 95% CI:0.01–0.87, *p* = 0.04, I^2^ = 96%). In a subgroup analysis, prolongation of pregnancy from study entry to delivery was statistically significant in cohort studies (mean difference 8.93 weeks, 95% CI:4.22–13.95, *p* = 0.00) but not in RCT studies (mean difference 0.37 weeks, 95%CI: (−0.06)−0.80, *p* = 0.09) (Figure 2).

#### NICU admission

**FIGURE 3:**
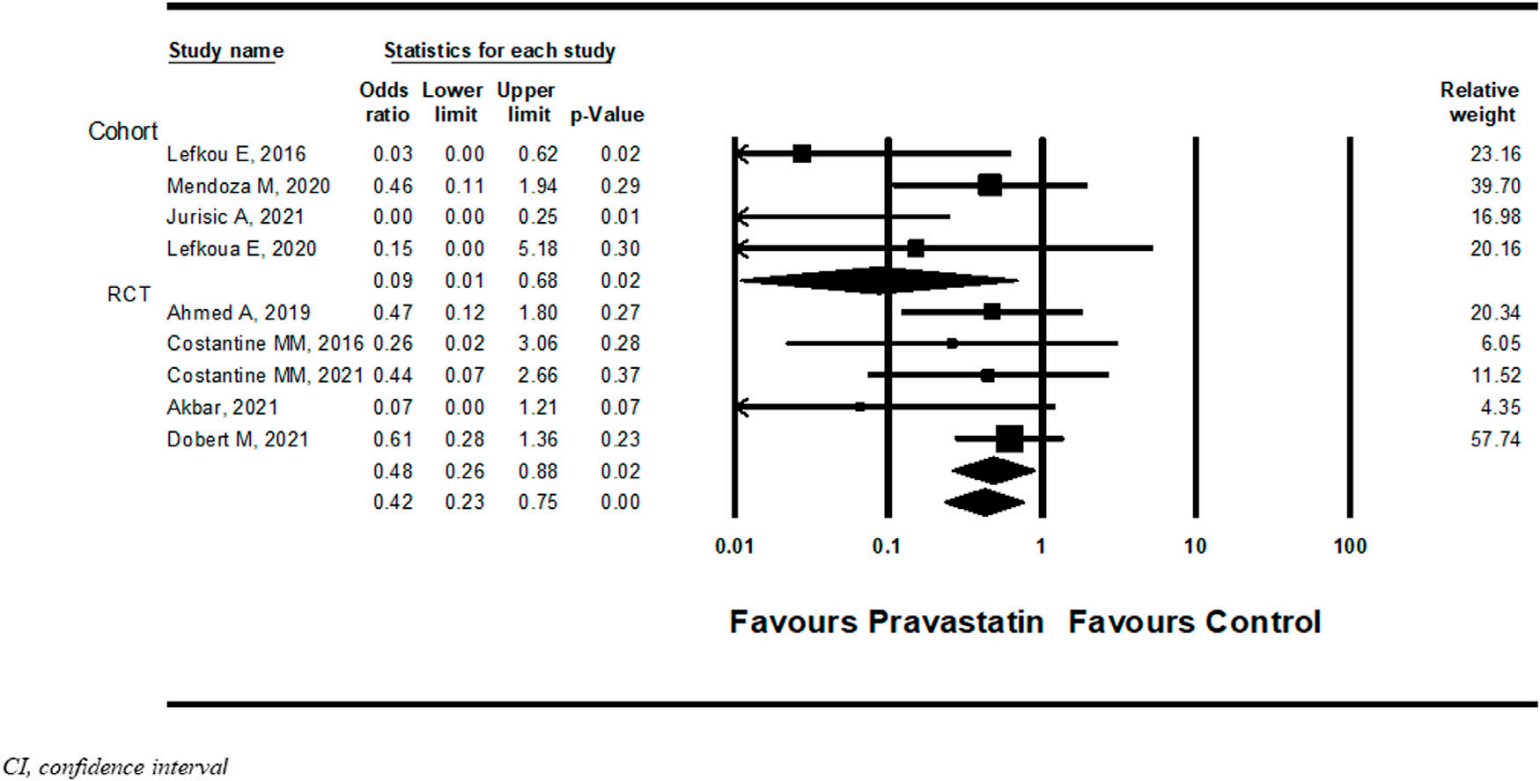
Meta-analysis results of the association of pravastatin treatment with admission to the neonatal intensive care unit.

Nine studies (Costantine et al., 2016; Lefkou et al., 2016; Ahmed et al., 2020; Lefkou et al., 2020; Mendoza et al., 2020; Akbar et al., 2021; Costantine et al., 2021; Dobert et al., 2021; Jurisic et al., 2021) examined NICU admission with or without pravastatin treatment. Pravastatin treatment was associated with a significantly decreased risk of NICU admission compared to women not treated with pravastatin (OR = 0.42, 95%CI: 0.23–0.75, *p* = 0.004, I^2^ = 25%). In a subgroup analysis, NICU admission was statistically significant both in cohort studies (OR 0.09, 95%CI:0.01–0.68, *p* = 0.02) and in RCT studies (OR 0.48, 95%CI:0.0.23–0.75, *p* = 0.02) (Figure 3).

#### Perinatal death

**FIGURE 4:**
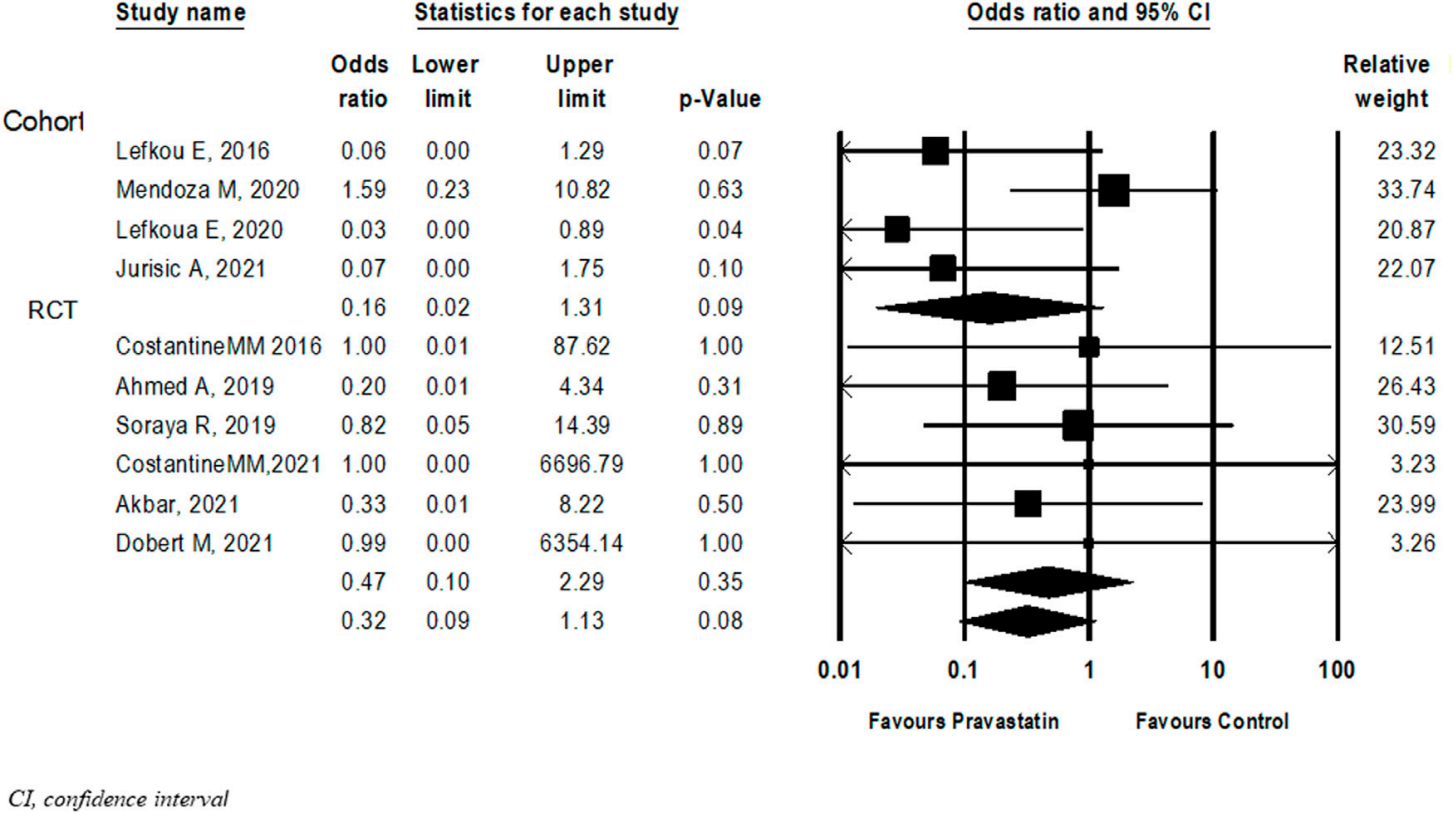
Meta-analysis results of Odds ratios for perinatal death following pravastatin treatment *versus* a control group.

Ten studies (Dobert et al., 2021; Costantine et al., 2016; Lefkou et al., 2016; Ahmed et al., 2020; Deviana et al., 2020; Lefkou et al., 2020; Mendoza et al., 2020; Akbar et al., 2021; Costantine et al., 2021; Jurisic et al., 2021) examined the association between pravastatin and perinatal death. Although statistical significance was not reached, not in the overall analysis and not in subgroup analysis according to study type, there was a trend towards a decrease in the risk of perinatal death in new-borns of women treated with pravastatin (OR = 0.32, 95%CI: 0.09–1.13, *p* = 0.08, I^2^ = 54%), (Figure 4).

#### Birth weight

**FIGURE 5:**
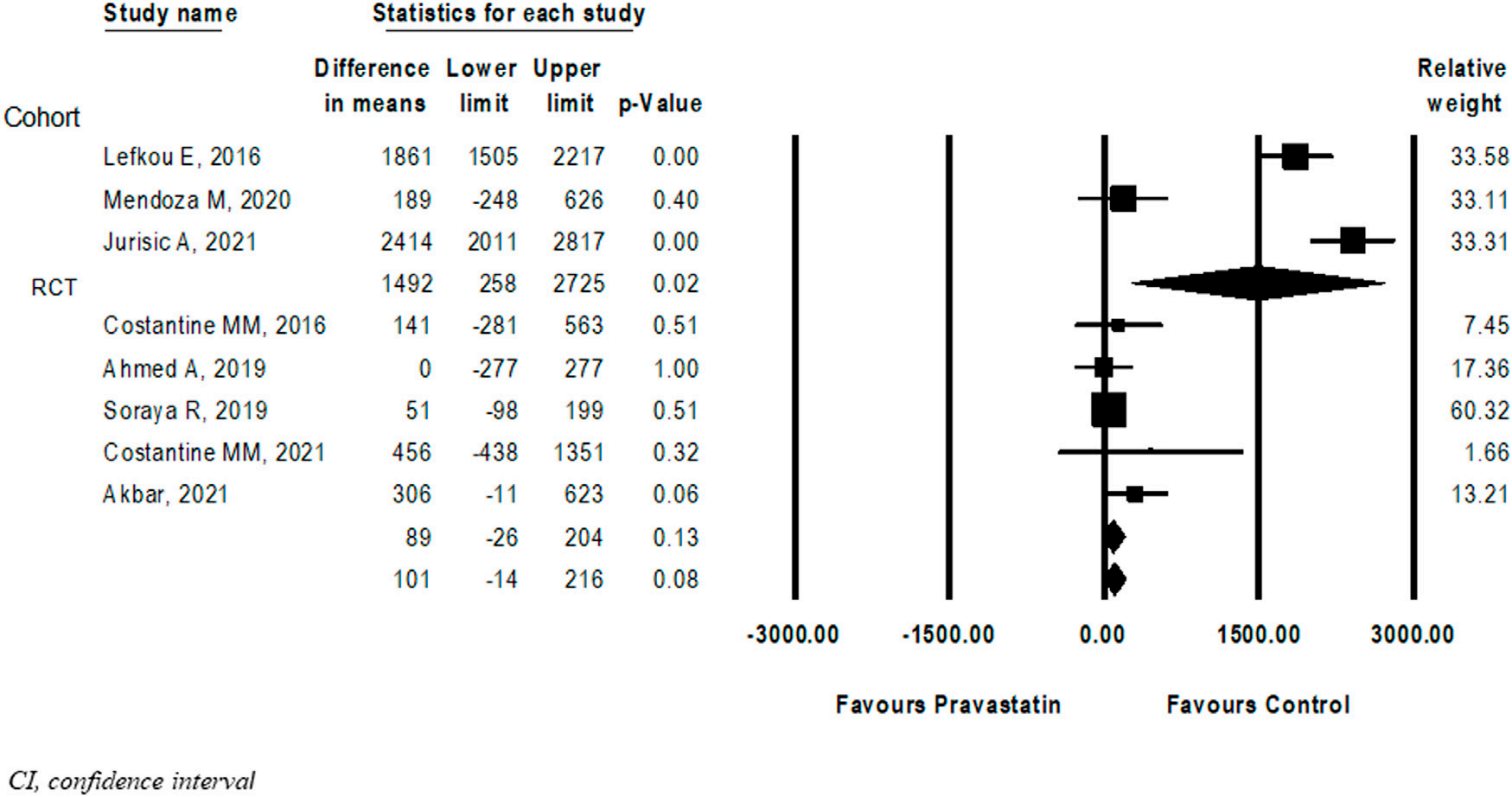
Meta-analysis results of the Differences in birth weight (grams) following pravastatin treatment *versus* a control group.

Eight (Costantine et al., 2016; Lefkou et al., 2016; Ahmed et al., 2020; Deviana et al., 2020; Lefkou et al., 2020; Mendoza et al., 2020; Costantine et al., 2021; Jurisic et al., 2021) studies compared birth weight between new-borns of women treated with pravastatin and those who were not. The difference in birth weight following pravastatin treatment was not statistically significant (Mean difference = 102 g, 95%CI: −14–212, *p* = 0.08, I^2^ = 96%). In a subgroup analysis, the difference in birthweight following pravastatin treatment was statistically significant in cohort studies (mean difference 1492g, 95%CI: 258–2,725, *p* = 0.02) but not in RCT studies (mean difference 89 g, 95%CI: (-26)-204, *p* = 0.13). (Figure 5).

#### Maternal outcomes

**FIGURE 6:**
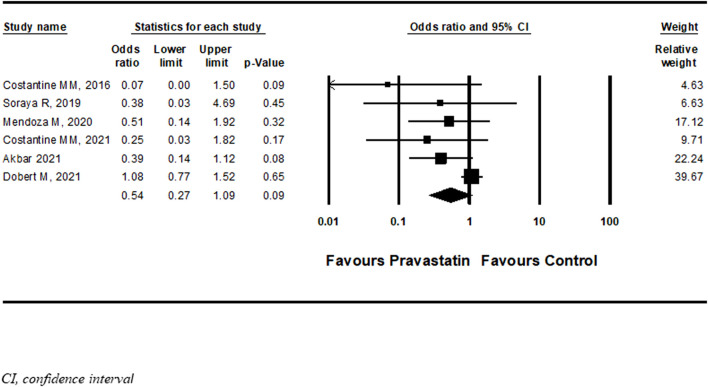
Meta-analysis results of the association of pravastatin treatment with new diagnoses of preeclampsia.

**FIGURE 7:**
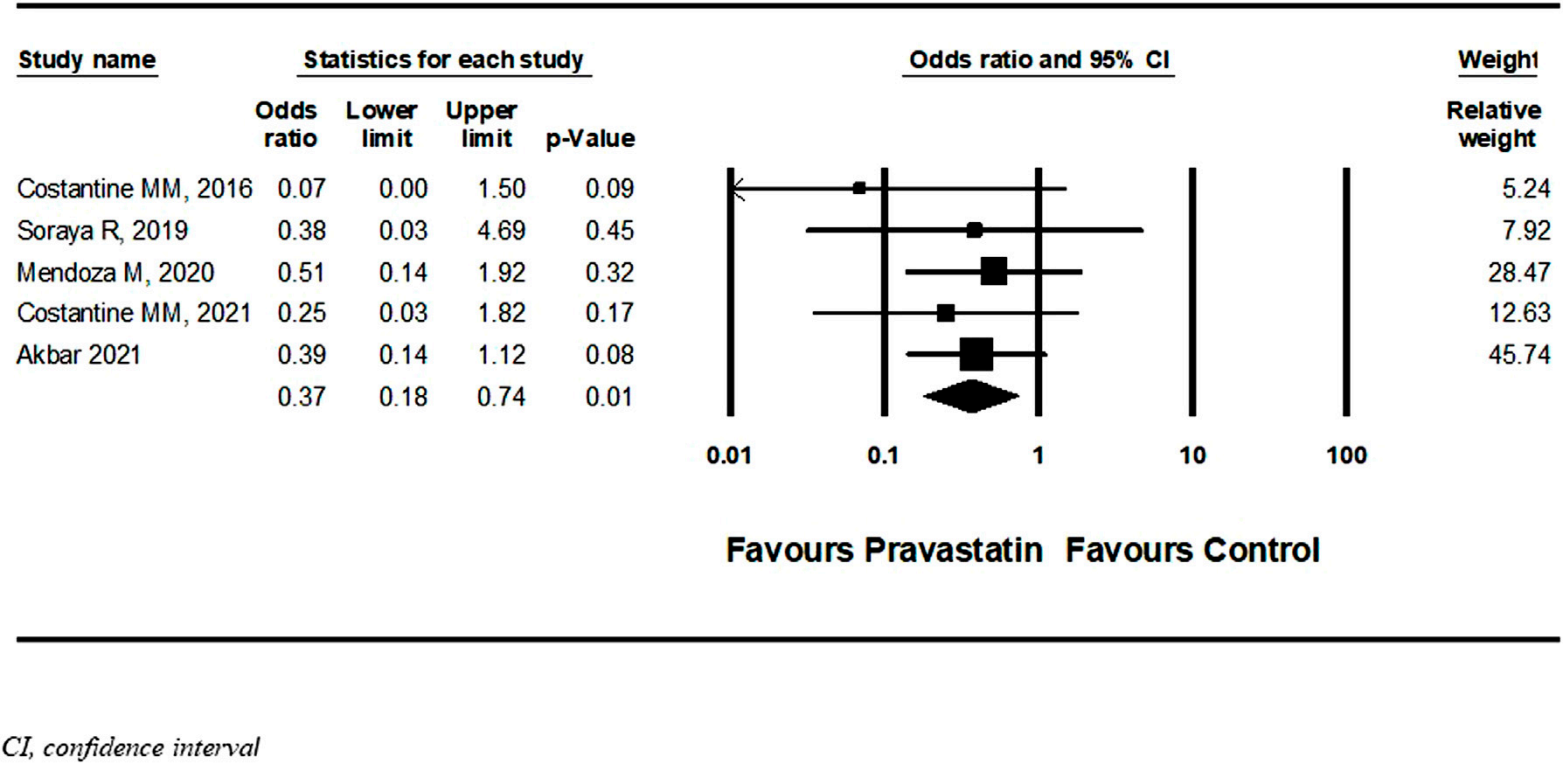
Sensitivity meta-analysis of the association of pravastatin treatment with new diagnoses of preeclampsia.

Six studies (Costantine et al., 2016; Deviana et al., 2020; Mendoza et al., 2020; Akbar et al., 2021; Costantine et al., 2021; Dobert et al., 2021) described a new diagnosis of PET after initiation of pravastatin treatment five of them were RCT and one cohort. Among women treated with pravastatin as opposed to those who were not treated, a nonsignificant decrease in further development of PET was demonstrated (OR = 0.54, 95%CI: 0.27–1.09, *p* = 0.09, I = 44%) (Figure 6) Dobert et al. (2021) specifically selected women in their late third trimester. A sensitivity analysis that excluded this study showed a significant decrease in the risk for developing PET (OR = 0.37, 95%CI:0.18–0.74, *p* = 0.01, I = 0%) (Figure 7). A further sensitivity analysis that excluded the cohort study of Mendoza et al. still showed a significant decrease in the risk for developing PET (OR = 0.32, 95%CI:0.14–0.73, *p* = 0.01) (Supplementary Figure S14).

#### Meta-regression and subgroup analysis

Due to the high heterogeneity found in the analyses, a meta-regression analysis was conducted. A meta-regression revealed a statistically significant association between early gestational age at initiation of pravastatin treatment and a decreased risk for further development of PET (R^2^ = 1) and NICU admission (R^2^ = 0.33). No associations were not found between higher pravastatin dose and prolongation of pregnancy from study entry to delivery, birth weight, perinatal death or NICU admission. No associations were not found between earlier gestational age at initiation of pravastatin treatment to prolongation of pregnancy from study entry to delivery, birth weight or perinatal death (Supplementary Figure S4–S13).

Subgroup analysis did not reveal a difference in the odds ratio for pregnancy prolongation, NICU admission, perinatal death and birth weight between studies that used pravastatin for primary vs secondary prevention (Supplementary Table S4).

## Discussion

### Main findings

In this meta-analysis and meta-regression, pravastatin usage, both for primary and secondary prevention of uteroplacental insufficiency disorders was associated with a significant prolongation of pregnancy, mean of 0.44 weeks, from study entry to delivery. Prematurity is the main cause of neonatal mortality and morbidity as well as late childhood morbidity. (Harrison and Goldenberg, 2016; Jurisic et al., 2021) Preventing preterm births has been delineated as one of the most urgent goals in current obstetrics. Among Pravastatin users, prolonging pregnancy has most probably resulted in the significant decrease observed in NICU admission, and the trends towards decreased perinatal death and increase in neonatal birth weight. In addition, in a sensitivity analysis that included women who had been treated in second trimester, new onset of PET was less common under pravastatin treatment.

### Comparison with existing literature

The similarities in pathophysiology and risk factors shared by uteroplacental insufficiency disorders and cardiovascular disease have prompted the search for treatments of uteroplacental insufficiency with different agents used to treat vascular disease (Rezai et al., 2021; Saif et al., 2021; Saito et al., 2021).The underlying mechanism of both conditions involves vascular endothelial dysfunction and endothelial inflammation. The American Heart Association included a history of PET as a risk factor for future cardiovascular disease (Mosca et al., 2011). This association might relate to the shared risk factors for cardiovascular diseases preceding pregnancy, such as obesity, hypertension, diabetes, and dyslipidaemia (Roberts and Cooper, 2001) or alternatively, to the metabolic and vascular changes that PET itself induces (Craici et al., 2008).

Numerous clinical studies have confirmed the multiple therapeutic benefits resulting from the pleiotropic effects of statins (Davignon, 2004; Ray and Cannon, 2005; Wang et al., 2008). Statins improve endothelial dysfunction by protecting vascular endothelium and stimulating its regeneration and its angiogenesis. The antioxidant action of statins relates to their antithrombotic and vasodilating activities and inhibition of free radical formation. The anti-inflammatory action of statins results from their impact on the immune response, which is expressed as increased blood level inflammatory markers and mediators. The latter include C-reactive protein, L-, E- and P-selectin, intercellular and vascular adhesion molecule (Davignon, 2004; Brownfoot et al., 2015; Zeisler et al., 2016). SFlt-1 is one of the key antiangiogenic circulating factors associated with the development of preeclampsia. Its levels are elevated in pregnant women weeks before the clinical onset of preeclampsia (Zeisler et al., 2016). Previous studies had demonstrated a reduction in levels of sFlt-1 by Hmox, both *in vitro* (Cudmore et al., 2007) and in mice models (Rezai et al., 2021). Hmox-1 as well as sFlt-1 pathways are regulated by Statins. Placental studies and a case series (Brownfoot et al., 2015) indicated that sFlt-1 levels decrease after statin therapy, but the clinical application of this finding is unclear. Our meta-analysis had four studies that investigated the pravastatin effect on sFlt-1, with contradicting results: Ahmed et al. (2020) reported a non-significant reduction in sFlt-1 levels. Mendoza et al. (2020) reported that in women with early-onset FGR, treatment with pravastatin was associated with significant improvement in sFlt-1/Placental Growth Factor (PIGF) ratio before and during pravastatin treatment. Akbar et al. (2021) reported a significant decrease in sFlt-1 levels before and after pravastatin treatment, and only Costantine et al. (2016) reported no difference at all in sFlt-1 in the umbilical cord between groups.

The safety profile of statin exposure during pregnancy is not well defined. United States Food and Drug Administration (FDA) labelled recommends against the use of statins during pregnancy, based on animal data showing teratogenic potential at high doses (Edison and Muenke, 2004). Therefore, the current practice encompasses the advice to discontinue statins when trying to conceive. However, due to subsequent case registries that did not demonstrate an association between congenital anomalies and statin exposure (Vahedian-Azimi et al., 2021), the FDA recently requested a revision to the information about pregnancy usage of the entire class of statins. The clinical benefits of statins in individuals with familial hypercholesterolemia or cardiovascular diseases should be considered, together with the growing evidence of statins’ potential benefit in preventing and treating uteroplacental insufficiency-associated disorders. Accordingly, their benefit may fairly overcome their controversial risk.

### Strengths and limitations

To the best of our knowledge, this is the largest and the only study to pool results of pravastatin for uteroplacental insufficiency-associated disorders. This meta-analysis included all available published data and conducted a systematic analysis according to accepted guidelines. Six RCT’s were included, and all ten studies described high treatment adherence.

As four studies included in this meta-analysis were retrospective, selection bias should be considered. We performed a quality assessment for the risk of bias using the NOS, which resulted in a low probability of selection bias. Due to the small numbers of studies and events, our results should be interpreted with caution, and further studies are required. The cohorts included in this meta-analysis were geographically diverse. This can potentially broaden the generalizability of our results.

The potential weakness of this analysis rests in the high heterogeneity among the studies, and the inclusion of only six RCTs’ with a small number of patients. The favourable outcomes of this meta-analysis may be driven by “cohort” studies and their inherited biases, as the women included in the control groups represented very specific populations. Yet, to address this heterogeneity, a sub-group analysis and random effect model was used. High heterogeneity was also viewed in dosage regimes, treatment indications and the lack of standardization. To address the high heterogeneity, we conducted a meta-regression for pravastatin dosage, study type and early vs. late initiation of pravastatin treatment. Although the analyses of perinatal death and birth weight showed trends toward better outcomes, these trends were not statistically significant, this might a be attributed to the small numbers of precipitants.

While the pathophysiology of uteroplacental dysfunction lies in inadequate trophoblast invasion in the early stages of pregnancy (Roberts and Cooper, 2001), all the studies that investigated pravastatin therapy started in the second to the third trimester and the largest study (Dobert et al., 2021) included only women in late third trimester which might downgrade the beneficial effect of the treatment, earlier initiation of therapy might yield different results from those described. Biomolecular markers, mainly sFlt1 or sFlt-1/PlGF ratio, were measured in few studies. In this meta-analysis we had decided not to address this issue as the reported results may be biased. The bias may be attributed to lack of standardization of measurements performed over various periods of time and to the exclusion of women who delivered after the first measurement from the analysis. In addition, no data were found in the literature on other potent statins such as atorvastatin and rosuvastatin (Stone et al., 2013).

In this analysis, we pooled studies that adjusted for confounders including pre-existing diabetes, a baseline score of biomolecule markers and laboratory together with studies that did not perform any adjustments, this (Jurisic et al., 2021), (Lefkou et al., 2016), (Mendoza et al., 2020) raises the possibility that our results may not reflect the true effect size and may be susceptible to sources of bias.

## Conclusion

This meta-analysis suggests that treatment with pravastatin for both primary and secondary prevention of uteroplacental insufficiency disorder may prolong pregnancy duration. An individualized assessment and a personalized approach are advised when pravastatin usage is considered.

## Data Availability

The original contributions presented in the study are included in the article/supplementary material, further inquiries can be directed to the corresponding author.
