# Supplementary material for: Pravastatin and placental insufficiency associated disorders: A systematic review and meta-analysis
Source: Front Pharmacol. 2022 Nov 9;13:1021548. doi: 10.3389/fphar.2022.1021548 (PMC9682185; doi:10.3389/fphar.2022.1021548)
Supplement: Supplementary file 1 [file DataSheet1.docx]

# Supplementary material

Table S1- **Checklist of Items to Include When Reporting A Systematic Review Involving a Meta-analysis**

| **Section and Topic** | **Item #** | **Checklist item** | **Location where item is reported** |
| --- | --- | --- | --- |
| **TITLE** | | |  |
| Title | 1 | Identify the report as a systematic review. | 1 |
| **ABSTRACT** | | |  |
| Abstract | 2 | See the PRISMA 2020 for Abstracts checklist. | 2-3 |
| **INTRODUCTION** | | |  |
| Rationale | 3 | Describe the rationale for the review in the context of existing knowledge. | 4-5 |
| Objectives | 4 | Provide an explicit statement of the objective(s) or question(s) the review addresses. | 5 |
| **METHODS** | | |  |
| Eligibility criteria | 5 | Specify the inclusion and exclusion criteria for the review and how studies were grouped for the syntheses. |  |
| Information sources | 6 | Specify all databases, registers, websites, organisations, reference lists and other sources searched or consulted to identify studies. Specify the date when each source was last searched or consulted. | 6-7 |
| Search strategy | 7 | Present the full search strategies for all databases, registers and websites, including any filters and limits used. | 6 |
| Selection process | 8 | Specify the methods used to decide whether a study met the inclusion criteria of the review, including how many reviewers screened each record and each report retrieved, whether they worked independently, and if applicable, details of automation tools used in the process. | 7 |
| Data collection process | 9 | Specify the methods used to collect data from reports, including how many reviewers collected data from each report, whether they worked independently, any processes for obtaining or confirming data from study investigators, and if applicable, details of automation tools used in the process. | 6-8 |
| Data items | 10a | List and define all outcomes for which data were sought. Specify whether all results that were compatible with each outcome domain in each study were sought (e.g. for all measures, time points, analyses), and if not, the methods used to decide which results to collect. | 7-8 |
|  | 10b | List and define all other variables for which data were sought (e.g. participant and intervention characteristics, funding sources). Describe any assumptions made about any missing or unclear information. | 7-8 |
| Study risk of bias assessment | 11 | Specify the methods used to assess risk of bias in the included studies, including details of the tool(s) used, how many reviewers assessed each study and whether they worked independently, and if applicable, details of automation tools used in the process. | 8-9 |
| Effect measures | 12 | Specify for each outcome the effect measure(s) (e.g. risk ratio, mean difference) used in the synthesis or presentation of results. |  |
| Synthesis methods | 13a | Describe the processes used to decide which studies were eligible for each synthesis (e.g. tabulating the study intervention characteristics and comparing against the planned groups for each synthesis (item #5)). | 8-9 |
|  | 13b | Describe any methods required to prepare the data for presentation or synthesis, such as handling of missing summary statistics, or data conversions. | 8 |
|  | 13c | Describe any methods used to tabulate or visually display results of individual studies and syntheses. | 8-9 |
|  | 13d | Describe any methods used to synthesize results and provide a rationale for the choice(s). If meta-analysis was performed, describe the model(s), method(s) to identify the presence and extent of statistical heterogeneity, and software package(s) used. | 8-9 |
|  | 13e | Describe any methods used to explore possible causes of heterogeneity among study results (e.g. subgroup analysis, meta-regression). | 8-9 |
|  | 13f | Describe any sensitivity analyses conducted to assess robustness of the synthesized results. | 9 |
| Reporting bias assessment | 14 | Describe any methods used to assess risk of bias due to missing results in a synthesis (arising from reporting biases). | 8-9 |
| Certainty assessment | 15 | Describe any methods used to assess certainty (or confidence) in the body of evidence for an outcome. | 8-9 |
| **RESULTS** | | |  |
| Study selection | 16a | Describe the results of the search and selection process, from the number of records identified in the search to the number of studies included in the review, ideally using a flow diagram. | 10 |
|  | 16b | Cite studies that might appear to meet the inclusion criteria, but which were excluded, and explain why they were excluded. | 10 |
| Study characteristics | 17 | Cite each included study and present its characteristics. | 11 |
| Risk of bias in studies | 18 | Present assessments of risk of bias for each included study. | 11+fig S1-S2 |
| Results of individual studies | 19 | For all outcomes, present, for each study: (a) summary statistics for each group (where appropriate) and (b) an effect estimate and its precision (e.g. confidence/credible interval), ideally using structured tables or plots. | 11-13 |
| Results of syntheses | 20a | For each synthesis, briefly summarise the characteristics and risk of bias among contributing studies. | 11-13 |
|  | 20b | Present results of all statistical syntheses conducted. If meta-analysis was done, present for each the summary estimate and its precision (e.g. confidence/credible interval) and measures of statistical heterogeneity. If comparing groups, describe the direction of the effect. | 11-13 |
|  | 20c | Present results of all investigations of possible causes of heterogeneity among study results. | 11-13 |
|  | 20d | Present results of all sensitivity analyses conducted to assess the robustness of the synthesized results. | 11-13+fig S3-S12 |
| Reporting biases | 21 | Present assessments of risk of bias due to missing results (arising from reporting biases) for each synthesis assessed. | 11 |
| Certainty of evidence | 22 | Present assessments of certainty (or confidence) in the body of evidence for each outcome assessed. | 11 |
| **DISCUSSION** | | |  |
| Discussion | 23a | Provide a general interpretation of the results in the context of other evidence. | 13 |
|  | 23b | Discuss any limitations of the evidence included in the review. | 15-16 |
|  | 23c | Discuss any limitations of the review processes used. | 15-16 |
|  | 23d | Discuss implications of the results for practice, policy, and future research. | 16-17 |
| **OTHER INFORMATION** | | |  |
| Registration and protocol | 24a | Provide registration information for the review, including register name and registration number, or state that the review was not registered. | 1 |
|  | 24b | Indicate where the review protocol can be accessed, or state that a protocol was not prepared. | 1 |
|  | 24c | Describe and explain any amendments to information provided at registration or in the protocol. | - |
| Support | 25 | Describe sources of financial or non-financial support for the review, and the role of the funders or sponsors in the review. | 1 |
| Competing interests | 26 | Declare any competing interests of review authors. | 1 |
| Availability of data, code and other materials | 27 | Report which of the following are publicly available and where they can be found: template data collection forms; data extracted from included studies; data used for all analyses; analytic code; any other materials used in the review. | Supplementary table S1, table 3 |

*Table S2- MOOSE Checklist for Meta-analyses of Observational Studies:*

| **Item No** | **Recommendation** | **Reported on Page No** |
| --- | --- | --- |
| Reporting of background should include | | |
| 1 | Problem definition | 5 |
| 2 | Hypothesis statement | - |
| 3 | Description of study outcome(s) | 7 |
| 4 | Type of exposure or intervention used | 6-7 |
| 5 | Type of study designs used | 6-7 |
| 6 | Study population | 6-7 |
| Reporting of search strategy should include | | |
| 7 | Qualifications of searchers (eg, librarians and investigators) | Title page |
| 8 | Search strategy, including time period included in the synthesis and key words | 5 , Fig 1 |
| 9 | Effort to include all available studies, including contact with authors | 6-7 |
| 10 | Databases and registries searched | 5-6 |
| 11 | Search software used, name and version, including special features used (eg, explosion) | 8 |
| 12 | Use of hand searching (eg, reference lists of obtained articles) | - |
| 13 | List of citations located and those excluded, including justification | 8-9, Table 1, Fig 1 |
| 14 | Method of addressing articles published in languages other than English | 5 |
| 15 | Method of handling abstracts and unpublished studies | 8 |
| 16 | Description of any contact with authors | 7 |
| Reporting of methods should include | | |
| 17 | Description of relevance or appropriateness of studies assembled for assessing the hypothesis to be tested | 8-9 |
| 18 | Rationale for the selection and coding of data (eg, sound clinical principles or convenience) | 8-9 |
| 19 | Documentation of how data were classified and coded (eg, multiple raters, blinding and interrater reliability) | 8-9 |
| 20 | Assessment of confounding (eg, comparability of cases and controls in studies where appropriate) | 7 |
| 21 | Assessment of study quality, including blinding of quality assessors, stratification or regression on possible predictors of study results | 7 |
| 22 | Assessment of heterogeneity | 7-8 |
| 23 | Description of statistical methods (eg, complete description of fixed or random effects models, justification of whether the chosen models account for predictors of study results, dose-response models, or cumulative meta-analysis) in sufficient detail to be replicated | 7-8 |
| 24 | Provision of appropriate tables and graphics | Tables 1, Figs 1-6, Figs S1-S12 |
| Reporting of results should include | | |
| 25 | Graphic summarizing individual study estimates and overall estimate | Figs 1-6, Figs S1-S12 |
| 26 | Table giving descriptive information for each study included | Table 1 |
| 27 | Results of sensitivity testing (eg, subgroup analysis) | Table S-S12 |
| 28 | Indication of statistical uncertainty of findings | - |

*Table S3-Search sequences that were used in the various databases.*

| Search sequence | Database |
| --- | --- |
| (((("pregnan*"[Title/Abstract] OR "reproduction"[Title/Abstract] OR "conception"[Title/Abstract] OR "prenatal development"[Title/Abstract] OR "Embryology"[Title/Abstract] OR "prenatal exposure"[Title/Abstract] OR "prenatal drug exposure"[Title/Abstract] OR "intrauterine drug exposure"[Title/Abstract] OR "maternal drug exposure"[Title/Abstract] OR "foetal drug exposure"[Title/Abstract] OR "fetus exposure"[Title/Abstract] OR "prenatal environment"[Title/Abstract] OR "reproductive interference"[Title/Abstract] OR "child bearing"[Title/Abstract] OR "childbearing" [Title/Abstract] OR "childbirth"[Title/Abstract] OR "gestation*"[Title/Abstract] OR "Gravidity"[Title/Abstract] OR "labor"[Title/Abstract] OR "labour"[Title/Abstract] OR "Parturient"[Title/Abstract] OR "trimester"[Title/Abstract] OR "midtrimester"[Title/Abstract] OR "maternal"[Title/Abstract] OR "fetus"[Title/Abstract] OR "prenatal disorder"[Title/Abstract] OR ("Pregnancy"[MeSH Terms] OR "Embryology"[MeSH Terms] OR "Pregnant Women"[MeSH Terms] OR "Gravidity"[MeSH Terms] OR "Gestational Age"[MeSH Terms] OR "Parity"[MeSH Terms] OR "Pregnancy Trimesters"[MeSH Terms])) OR "pregnan*"[Title/Abstract] OR "[Title/Abstract] OR "prenatal development"[Title/Abstract] OR "embryology"[Title/Abstract] OR "prenatal exposure"[Title/Abstract] OR "prenatal drug exposure"[Title/Abstract] OR "intrauterine drug exposure"[Title/Abstract] OR "maternal drug exposure"[Title/Abstract] OR "foetal drug exposure"[Title/Abstract] OR "fetus exposure"[Title/Abstract] OR "prenatal environment"[Title/Abstract] OR "reproductive interference"[Title/Abstract] OR "child bearing"[Title/Abstract] OR "childbearing"[Title/Abstract] OR "childbirth"[Title/Abstract] OR "gestation*"[Title/Abstract] OR "gravidity"[Title/Abstract] OR "labor"[Title/Abstract] OR "labour"[Title/Abstract] OR "Parturient"[Title/Abstract] OR "trimester"[Title/Abstract] OR "midtrimester"[Title/Abstract] OR "maternal"[Title/Abstract] OR "fetus"[Title/Abstract] OR "prenatal disorder"[Title/Abstract]AND ("Hydroxymethylglutaryl coenzyme A reductase inhibitor"[Title/Abstract] OR "hydroxymethylglutaryl coa reductase inhibitors"[Title/Abstract] OR "hydroxymethylglutaryl coa reductase inhibitors"[Title/Abstract] OR "hmg coa reductase inhibitors"[Title/Abstract] OR "HMG CoA reductase inhibitor"[Title/Abstract] OR "hmg coa reductase inhibitors"[Title/Abstract] OR "hmg coenzyme a reductase inhibitor"[Title/Abstract] OR "anticholesteremic agent"[Title/Abstract] OR "hypocholesterolemic agent"[Title/Abstract] OR "statin*"[Title/Abstract] OR "vastatin"[Title/Abstract] OR "simvastatin"[Title/Abstract] OR "pitavastatin"[Title/Abstract] OR "lovastatin"[Title/Abstract] OR "Fluvastatin" [Title/Abstract] OR "pravastatin"[Title/Abstract] OR "atorvastatin"[Title/Abstract] OR "rosuvastatin"[Title/Abstract] OR "cerivastatin"[Title/Abstract] OR "compactin" [Title/Abstract] OR "crilvastatin"[Title/Abstract] OR "dalvastatin"[Title/Abstract] OR "Mevinolin"[Title/Abstract] OR "mevinolinic acid"[Title/Abstract] OR "monacolin" [Title/Abstract] OR ("hydroxymethylglutaryl coa reductase inhibitors"[MeSH Terms] OR "hydroxymethylglutaryl coa reductase inhibitors"[Pharmacological Action]))) NOT ("animals"[MeSH Terms] NOT "humans"[MeSH Terms])) NOT ("Systematic Review"[Publication Type] OR "meta-analysis"[Publication Type] OR "Review"[Publication Type] OR "Editorial"[Publication Type] OR "Comment"[Publication Type])) NOT "animals"[MeSH Terms] NOT "humans"[MeSH Terms] | PubMed |
| ((((pregnan*:ti,ab,kw OR reproduction:ti,ab,kw OR conception:ti,ab,kw OR 'prenatal development':ti,ab,kw OR embryology:ti,ab,kw OR 'prenatal exposure':ti,ab,kw OR 'prenatal drug exposure':ti,ab,kw OR 'intrauterine drug exposure':ti,ab,kw OR 'maternal drug exposure':ti,ab,kw OR 'foetal drug exposure':ti,ab,kw OR 'fetus exposure':ti,ab,kw OR 'prenatal environment':ti,ab,kw OR 'reproductive interference':ti,ab,kw OR 'child bearing':ti,ab,kw OR childbearing:ti,ab,kw OR childbirth:ti,ab,kw OR gestation*:ti,ab,kw OR gravidity:ti,ab,kw OR labor:ti,ab,kw OR labour:ti,ab,kw OR parturient:ti,ab,kw OR trimester:ti,ab,kw OR midtrimester:ti,ab,kw OR maternal:ti,ab,kw OR fetus:ti,ab,kw OR 'prenatal disorder':ti,ab,kw) OR ('pregnancy'/exp OR 'prenatal development'/exp OR 'prenatal disorder'/exp OR 'pregnant woman'/exp)) AND (('hydroxymethylglutaryl coenzyme a reductase inhibitor':ti,ab,kw OR 'hydroxymethylglutaryl coa reductase inhibitors':ti,ab,kw OR 'hydroxymethylglutaryl-coa reductase inhibitors':ti,ab,kw OR 'hmg-coa-reductase inhibitors':ti,ab,kw OR 'hmg coa reductase inhibitor':ti,ab,kw OR 'hmg coa reductase inhibitors':ti,ab,kw OR 'hmg coenzyme a reductase inhibitor':ti,ab,kw OR 'anticholesteremic agent':ti,ab,kw OR 'hypocholesterolemic agent':ti,ab,kw OR statin*:ti,ab,kw OR vastatin:ti,ab,kw OR simvastatin:ti,ab,kw OR pitavastatin:ti,ab,kw OR lovastatin:ti,ab,kw OR fluvastatin:ti,ab,kw OR pravastatin:ti,ab,kw OR atorvastatin:ti,ab,kw OR rosuvastatin:ti,ab,kw OR bervastatin:ti,ab,kw OR cerivastatin:ti,ab,kw OR compactin:ti,ab,kw OR crilvastatin:ti,ab,kw OR dalvastatin:ti,ab,kw OR mevinolin:ti,ab,kw OR 'mevinolinic acid':ti,ab,kw OR monacolin:ti,ab,kw) OR 'hydroxymethylglutaryl coenzyme a reductase inhibitor'/exp)) NOT ('animals'/exp NOT 'humans'/exp)) NOT ('review'/exp OR 'editorial'/exp OR 'note'/exp OR 'conference review'/exp OR 'systematic review'/exp OR 'meta analysis'/exp) AND [2020-2021]/py | Embase |
| obstetric OR obstetrical OR pregnant OR pregnancy OR reproduction OR conception OR "prenatal development" OR embryology OR "prenatal exposure" OR "prenatal drug exposure" OR "intrauterine drug exposure" OR "maternal drug exposure"OR "foetal drug exposure" OR "fetus exposure" OR "prenatal environment" OR "reproductive interference" OR "child bearing" OR childbearing OR childbirth OR gestation OR gravidity \| "Hydroxymethylglutaryl coenzyme A reductase inhibitor" OR "hydroxymethylglutaryl coa reductase inhibitors" OR "Hydroxymethylglutaryl-CoA reductase inhibitors" OR "HMG-CoA-reductase inhibitors" OR "HMG CoA reductase inhibitor" OR "HMG CoA reductase inhibitors" OR "hmg coenzyme a reductase inhibitor" OR "anticholesteremic agent" OR "hypocholesterolemic agent" OR statin OR statins OR vastatin OR simvastatin OR pitavastatin OR lovastatin OR Fluvastatin OR pravastatin OR atorvastatin OR rosuvastatin OR bervastatin OR cerivastatin OR compactin OR crilvastatin OR dalvastatin OR Mevinolin OR "mevinolinic acid" OR monacolin. | Clinical trials |
| pregnan* OR reproduction OR conception OR "prenatal development" OR embryology OR "prenatal exposure" OR "prenatal drug exposure" OR "intrauterine drug exposure" OR "maternal drug exposure" OR "foetal drug exposure" OR "fetus exposure" OR "prenatal environment" OR "reproductive interference" OR "child bearing" OR childbearing OR childbirth OR gestation* OR gravidity OR labor OR labour OR Parturient OR trimester OR midtrimester OR maternal OR fetus OR "prenatal disorder"):ti,ab,kw AND ("Hydroxymethylglutaryl coenzyme A reductase inhibitor" OR "hydroxymethylglutaryl coa reductase inhibitors" OR "Hydroxymethylglutaryl-CoA reductase inhibitors" OR "HMG-CoA-reductase inhibitors" OR "HMG CoA reductase inhibitor" OR "HMG CoA reductase inhibitors" OR "hmg coenzyme a reductase inhibitor" OR "anticholesteremic agent" OR "hypocholesterolemic agent" OR statin* OR vastatin OR simvastatin OR pitavastatin OR lovastatin OR Fluvastatin OR pravastatin OR atorvastatin OR rosuvastatin OR bervastatin OR cerivastatin OR compactin OR crilvastatin OR dalvastatin OR Mevinolin OR "mevinolinic acid" OR monacolin):ti,ab,kw (Word variations have been searched)) | Cochrane |

Figure S 1-Risk of bias summary for the cohort studies, according to the authors’ assessments.

| **Newcastle-Ottawa Quality Assessment Scale (NOS)** | | | | |
| --- | --- | --- | --- | --- |
| **NOS score** | **Outcome** | **Comparability** | **Selection** | **Study, year** |
| 7 | *** | * | *** | Lefkou et al, 2016 |
| 7 | *** | * | *** | Lefkou et al, 2020 |
| 9 | *** | ** | **** | Mendoza et al, 2020 |
| 7 | *** | * | *** | Jurisic et al, 2020 |

Figure S2-Risk of bias summary for the randomized controlled trials, according to the authors’ assessments.


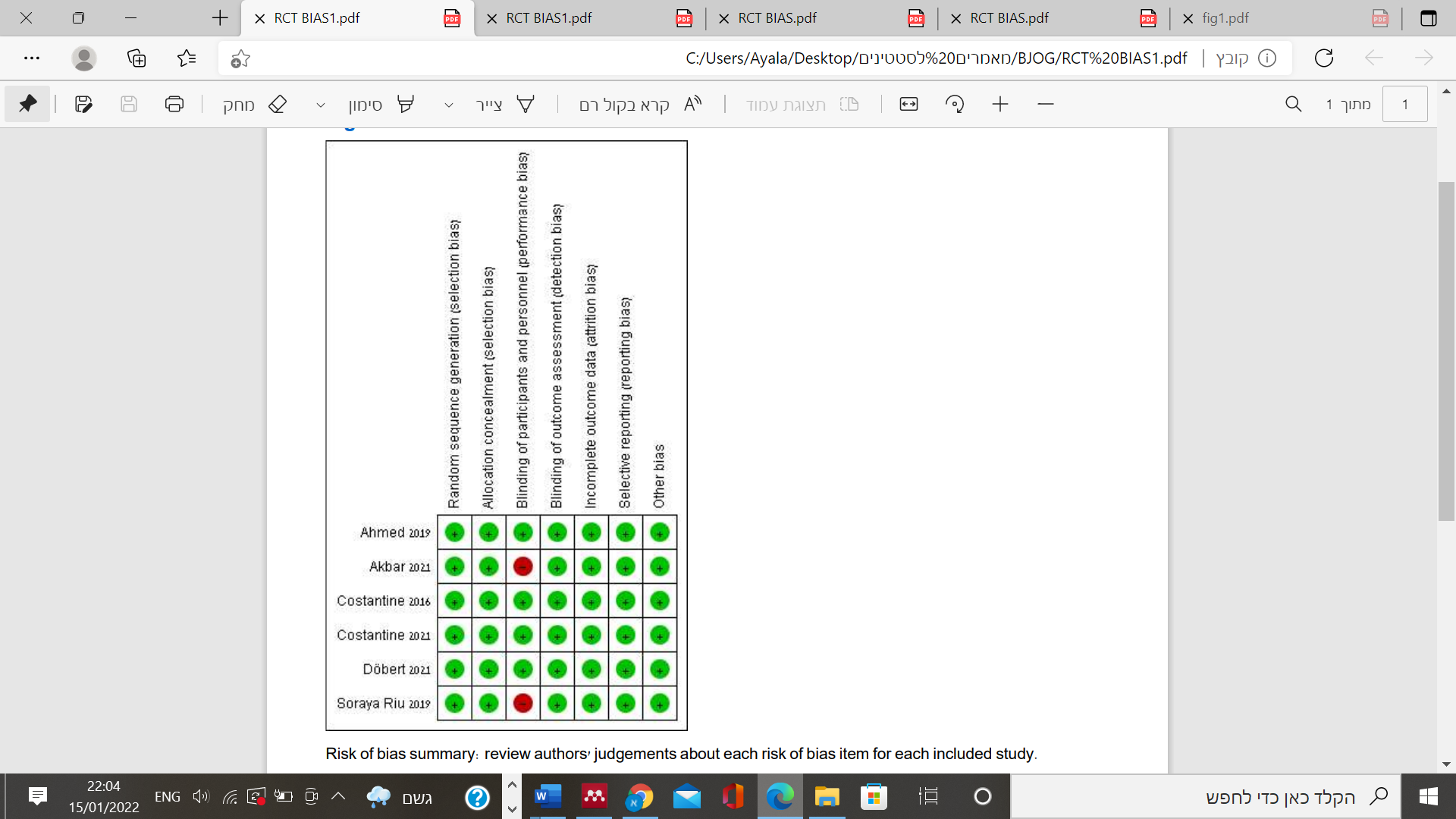


**Meta-regression**

Figure S3-Risk of bias summary graph

Figure S4- Meta-regression analysis results of the correlation between Pravastatin dosage(mg) and prolongation of pregnancy (weeks)

Figure S5- Meta-regression analysis results of the correlation between Pravastatin dosage (mg) and admission to the neonatal intensive care unit

Figure S6- Meta-regression analysis results of the correlation between Pravastatin dosage(mg) and perinatal death

Figure S7- Meta-regression analysis results of the correlation between Pravastatin dosage (mg) and birth weight (gr)

Figure S8- Meta-regression analysis results of the correlation between mean gestational age at the initiation of Pravastatin treatment (weeks) and prolongation of pregnancy (weeks).

Figure S9- Meta-regression analysis results of the correlation between mean gestational age at the initiation of Pravastatin treatment (weeks) and admission to neonatal intensive care unit

Figure S10- Meta-regression analysis results of the correlation between mean gestational age at the initiation of Pravastatin treatment (weeks) and perinatal death

*Figure S11- Meta-regression analysis results of the correlation between gestational age at initiation of Pravastatin treatment (weeks) with birth weight (gr)*

Figure S12- Meta-regression analysis results of the correlation between Pravastatin dosage (mg) and risk for the development of preeclampsia

*Figure S13- Meta-regression analysis results of the correlation of* *gestational age at initiation of Pravastatin treatment (weeks) with* *risk for the development of preeclampsia*

*Figure S14: Sensitivity meta-analysis results of the association of pravastatin treatment with new diagnoses of preeclampsia in RCT*

**

*Table S4*: *Comparison between studies that used pravastatin for treatment compared to those that used pravastatin for prevention*

| Treatment (95%CI) | Prevention (95%CI) | Outcome |
| --- | --- | --- |
| 5.2 weeks 95%CI:0.1-10.4, p=0.05 | 4.4 weeks 95%CI: -2.2-10.9, I2=87%, p=0.19 | *Pregnancy prolongation (mean difference, weeks)* |
| OR=0.34 95%CI: 0.13-0.85, I2=2%, p=0.02 | OR=0.25 95%CI: 0.07-0.86, I2=46%, p=0.03 | *NICU admission (Odd ratio)* |
| OR=0.21 95%CI:0.03-1.50, I2, p=0.12=49 | OR=0.37 95%CI:0.07-1.85, I2=0, p=0.23 | *Perinatal death  (Odd ratio)* |
| 683 grams 95%CI: -513-1879, I2=97%, p=0.26 | 674 grams 95%CI: -150-1499, I2=96%, p=0.11 | *Birth weight (grams)* |
